# Adherence to guidelines in the follow-up of non-muscle-invasive bladder cancer among urology trainers and trainees in Jordan: a cross-sectional study

**DOI:** 10.1097/MS9.0000000000000413

**Published:** 2023-04-04

**Authors:** Rami Al-Azab, Mohammad Al-Zubi, Saddam Al Demour, Suad Khaled Al-Jamal, Lobana Nabeel Mahdawi, Salsabeel Saleh Al-Omari, Rania Rasmi Banibakr, Luma Ali Alhallaq, Yaseen Abdelqader Yaseen Asa’d, Wasan Omar Rjoub

**Affiliations:** a Department of Surgery and Urology, School of Medicine, Jordan University of Science and Technology; b Division of Urology, School of Medicine, Department of Surgery; c Faculty of Medicine, Yarmouk University, Irbid; d Division of Urology, School of Medicine, Department of Special Surgery, The University of Jordan, Amman; e Faculty of Medicine, Hashemite University, Zarqa, Jordan

**Keywords:** bladder cancer, follow-up adherence, staging

## Abstract

To assess the clinical practice in the follow-up of non-muscle-invasive bladder cancer (NMIBC) among urology specialists (trainers) and residents (trainees) in Jordan.

**Methods:**

An electronic questionnaire containing, in addition to demographic data, four questions regarding the follow-up of NMIBC was sent by e-mail to 115 urologists (53 residents and 62 specialists) selected randomly by stratified random sampling from different clinical institutions, 105 of them were returned complete.

**Results:**

In all, 105 out of 115 (91%) questionnaires were returned complete. All of the candidates are male. For low-risk NMIBC follow-up, 46 of the specialists (79%) and 35 of the trainees (74%) decided to do a follow-up cystoscopy at 3 months after diagnosis, followed by a check cystoscopy 9 months later than yearly, while for high-risk patients, all of the specialists and 45 of the trainees (96%) decide to do a check cystoscopy every 3 months in the first 2 years after diagnosis. For upper tract follow-up in high-risk NMIBC, all of the urologists in the survey (specialists and trainees) routinely perform upper tract imaging in the form of a computed tomography scan with contrast in the first year after diagnosis. On the other hand, in the follow-up of the upper urinary tract in low-risk NMIBC, 16 of the trainees (34%) and 19 of the specialists (33%) still perform a yearly scan.

**Conclusion:**

Because of the high recurrence rate for NMIBC, this raises the importance of adherence to guidelines in the follow-up for these patients and, at the same time to avoid overdoing unnecessary cystoscopies or upper tract scans.

## Introduction

Highlights

- Bladder cancer is considered the most common urogenital cancer worldwide.
- The main staging system used for bladder cancer is the American Joint Committee on Cancer (AJCC) TNM [tumor (T), nodes (N), and metastases (M)] system.
- Multifactorial and complex causes are behind the pathogenesis of bladder cancer development, including different environmental, genetic, or molecular factors.

Bladder cancer is considered the most common urogenital cancer worldwide^1^, with the highest incidence rates seen in North America, Southern and Western Europe, and in certain countries in Northern Africa or Western Asia^2^. Men are four times more susceptible to developing bladder cancer than women, with an incidence of 2.4/100 000 among women compared to 9.6/100 000 among men^3^. Bladder cancer is a disease of the elderly, with ∼80% of cases diagnosed in adults over 65^3^.

Multifactorial and complex causes are behind the pathogenesis of bladder cancer development, including different environmental, genetic, or molecular factors^4^. Most of the cases are attributed to genetic or epigenetic alteration of tumor suppressor genes, growth factor receptors, and DNA repair genes. A mutation in *FGFR3* and *HRAS* is found in 70 and 30%, respectively, of bladder cancer cases^5^.

Cigarette smoking is considered the leading cause of bladder cancer; about 50% of cases of bladder cancer in developed countries are related to smoking^6^. Other strong risk factors are exposure to certain drugs such as chemotherapy, especially cephalosporine, alcohol, obesity, and arsenic^6^. Also, patients who have chronic urinary tract infections, chronic use of urinary catheters, and bladder stones have a higher risk for bladder cancer^7^. The main staging system used for bladder cancer is the American Joint Committee on Cancer (AJCC) TNM system^8^, which classifies tumors based on local invasive (T), lymph node metastasis (N), and distance metastasis (M)^9^. Accurate bladder cancer staging is essential to select the best therapeutic plan. The pathologic stage (pTNM) provides the most accurate information to evaluate the treatment plan and prognosis. This staging classification is still one of the strongest indicators of the outcome of bladder cancer^10^. Approximately 70% of patients present with superficial tumors (Ta, T1, carcinoma in situ), whereas muscle-invasive disease (T2–4) presents in 3 out of 10 with a high risk of metastases to distance region. Moreover, the recurrence rate of superficial tumors is between 50% and 70%, with ∼10–20% risk of progressing to muscle-invasive disease^11^. For that, close follow-up of non-muscle-invasive bladder cancer (NMIBC) by doing regular check cystoscopy and upper tract imaging, if needed, is important. So, in this research, we studied the adherence of urology trainers and trainees to guidelines in NMIBC follow-up for the first 2 years after diagnosis. The importance of this study is to show how important to choose the correct follow-up plan for NMIBC patients so as to detect recurrence early in these patients and, at the same time, avoid doing unnecessary tests and procedures. This study was written in accordance with the STROCSS (strengthening the reporting of cohort, cross-sectional and case–control studies in surgery) guidelines^12^.

## Materials and Methods

### Study design

This is an analytic cross-sectional study to see the adherence of our specialists (trainers) and residents (trainees) in Jordan to guidelines in the follow-up of NMIBC in the first 2 years after diagnosis. The electronic questionnaire utilizing Google Forms consists of two parts: the first one is for demographic data (age, sex, years of experience), and the second part contains four questions to assess urologists’ practice in the follow-up of NMIBC and upper urinary tract in the first 2 years after diagnosis of bladder cancer. Questionnaires were sent by e-mail, from May 2022 to July 2022, to 115 urologists, 53 of them were residents (trainees) and 62 specialists (trainers). Candidates were randomly selected (by stratified random sampling according to registration number in Jordan Medical Association) from different clinical hospitals, including university hospitals, military hospitals, and private hospitals, in addition to the Ministry of Health. One hundred five of the questionnaires were returned complete by e-mail (58 specialists and 47 residents), while the remaining 10 (4 specialists and 6 residents) were incomplete, so they were excluded from the study. The study has been registered with https://www.researchregistry.com, UIN: researchregistry8717.

### Questionnaire

The second part of the questionnaire consists of four questions selected after a deep discussion with three expert urology consultants in Jordan to measure the knowledge and adherence to guidelines in the follow-up of NMIBC in the first 2 years after diagnosis.

### Statistics

Data entry and analysis were performed using the SPSS statistical package (version 20). Frequency and percentage tables were created to show the descriptive statistics. At the same time, mean and SD were used to express the age and year of experience for both the specialist and trainees. Categorical data were analyzed using the *χ* ^2^ test. A *P* value of less than 0.05 was considered statistically significant.

## Results

A total of 105 out of 115 (91%) questionnaires were returned complete, 47 (45%) by trainees and 58 (55%) by specialists. The mean age of urologists in the study was 36.3 years for specialists and 29.7 for residents. All of the candidates in the study are male (100%). Regarding years of experience for the urologists in this survey, 31% have more than 10 years of experience in the field, while 40% have 5 years or less experience in urology (Table 1 shows the demographic characteristics of the sample). As low-risk NMIBC patients have a lower risk of recurrence than high-risk patients, 46 of the specialists (79%) and 35 of the trainees (74%) decided to follow-up cystoscopy at 3 months after diagnosis, followed by check cystoscopy 9 months later, and then yearly. However, 12 of the specialists (21%) and 12 of the trainees (26%) preferred to check the bladder by cystoscopy more frequently, every 3 months after NMIBC diagnosis for 2 years after diagnosis with a *P* value of 0.643 (nonsignificant) between both groups (trainers and trainees). While for high-risk patients follow-up, all of the specialists and 45 of the trainees (96%) do check cystoscopy every 3 months in the first 2 years after diagnosis with also nonsignificant *P* value (*P*=0.198) between both groups in the follow-up of high-risk NMIBC. As high-risk NMIBC can increase the risk of upper urinary tract malignancy, all of the urologists in the survey (specialists and trainees) routinely performed upper tract imaging in the form of computed tomography scan with contrast in the first year after diagnosis and then every 2 years to follow the upper tract. In general, urologists had better adherence to the aforementioned guidelines than residents due to their experience. When once asked about the follow-up of the upper urinary tract in low-risk NMIBC, 16 trainees (34%) and 19 specialists (33%) still perform a yearly scan for upper tract follow-up (*P*=1). As seen in Table 2, there is no statistically significant difference in the clinical follow-up of low and high-risk NMIBC patients for both cystoscopy and upper urinary tract follow-up scans between trainers and trainees.

**Table 1:** Demographic data for urologists (specialists and residents)

	Count	Frequency (%)
Specialists	58	55
Residents	47	45
Years of experience		
0–5	42	40
6–10	30	29
>10	33	31
Male sex	105	100
Age		
26–30	17	16.2
31–35	32	30.5
36–40	19	18.1
41–45	17	16.2
>45	20	19

**Table 2:** Answers for NMIBC follow-up questions (for both specialists and residents) and *P* values

		Specialists		Residents		
Question	Answer	Count	Frequency (%)	Count	Frequency (%)	*P*
For low-risk NMIBC, will do check cystoscopy at 3, 12, and yearly	Yes	46	79	35	74	0.643
For high-risk NMIBC, will do check cystoscopy every 3 months in the first 2 years after diagnosis	Yes	58	100	45	96	0.198
For low-risk NMIBC, will do upper tract imaging every year	Yes	19	33	16	34	1
For high-risk NMIBC, will do upper tract imaging every year	Yes	58	100	47	100	–

NMIBC, non-muscle-invasive bladder cancer.

## Discussion

In regard to the management of NMIBC, the high-quality transurethral resection of bladder tumor (TURBT) is very important in diagnosing, staging, and managing NMIBC. By using it as initial treatment, we resect the tumor completely and sample the detrusor muscle below it. The use of some techniques like blue light cystoscopy and narrow band imaging will increase tumor detection, thus decreasing the recurrence risk^13^.

For therapeutic and prophylactic cases, we use adjuvant intravesical chemotherapy and immunotherapy. We use single instillation of chemotherapy post-TURBT to decrease malignant cells and recurrence rates, which is recommended for intermediate-risk NMIBC, but its usage is unclear in those with high risk. BCG (Bacillus Calmette-Guérin) therapy is given every week for 6 weeks, followed by 3 years of maintenance for high-risk NMIBC, but 1 year maintenance is enough for intermediate-risk NMIBC^13^,^14^.

In cases of large-volume diffuse and endoscopically unresectable NMIBC upfront, radical cystectomy is recommended. It is also used for patients with high-grade, recurrent NMIBC^15^.

Follow-up for NMIBC is not an easy issue for doctors because it has flexible corners that can change with the situation of the case. Although it does not have significant evidence, some of them are difficult to do in clinics and costly procedures; it remains to have an important role in detecting recurrences and progression. The main recommendations go with using cystoscopy, upper urinary tract imaging, and to a lesser extent, urine cytology. New studies and experiments go with urine markers which are thought to optimize the follow-up done in the traditional technique, especially in the point that it showed very high negative predictive values for recurrences in the follow-up of NMIBC, especially high-grade recurrences^16^,^17^. Now those methods are not performed randomly; it is an organized process in which for low-risk NMIBC, cystoscopy at 3 months, 12 months, then annually is sufficient, and the upper tract evaluation or cytology is done only at diagnosis. On the other hand, for high-risk tumors, more intense follow-up is needed; cystoscopy is done every 3 months for 2 years, 6 months for 2 years, and then annually. Cytology, to study the bladder cells, is performed at frequent intervals, whereas the imaging is done for upper tract evaluation 1 year after the treatment and then every 2 years. Urine markers are mentioned above; although it is approved that it is more sensitive than cytology and significantly reduces the patient burden, the main impediment of it is its high cost^18^!

NMIBC can be classified into three risk groups: low, intermediate, and high-risk cancer. This classification detects whether cancer will progress or regress, which will guide the treatment and follow-up of NMIBC. The most important factor in this classification is the grade of cancer^19^. The grade of bladder cancer is divided into three groups: papillary urothelial neoplasia of low malignant potential, low grade, and high grade^13^,^14^.

According to the Modified Canadian Urological Association risk stratification system^20^:

1. Low risks include primary, solitary, Ta, low grade, size less than 3 cm, and no carcinoma in situ (CIS).
2. Intermediate risk includes any of the following:
  - recurrent, multifocal, and/or large size (>3 cm) low-grade Ta;
  - primary, solitary, and small (<3 cm) high-grade Ta.
3. High risk includes any of the following:
  - T1 tumor;
  - recurrent, multiple, and size (≥3 cm) high-grade Ta;
  - presence of CIS (primary or concomitant);
  - multiple, recurrent and large sizes (>3 cm) Ta with grades G1 and G2 tumors.

Following TURBT, both cytology and cystoscopy are recommended after 3 months for all risk groups. Recommendations for follow-up after that depend on the risk stratification. In low-risk tumors, a cystoscopy every year is enough. High risk is followed up by cystoscopy every 3 months for 2 years, twice annually for another 2 years, then every year with adjunct cytology and upper urinary tract imaging at 1 year, and then every 2 years. The intermediate-risk group should be followed more intense than the low-risk group but to a lesser extent than the high-risk group^18^.

## Conclusion

The high recurrence rate of NMIBC warrants close follow-up for the patients and, at the same time to avoid overdoing unnecessary follow-up cystoscopies or upper tract scans; this raises the importance of doing regular meetings involving both trainees and trainers to classify NMIBC patients into high, intermediate, and low-risk groups and put correct follow-up plan for each patient, by doing this we can guarantee correct follow-up for each patient. Moreover, our study shows that urology specialists are more adherent to tested guidelines than residents due to their high years of experience in the field.

## Ethical approval

The ethical approval for conducting this study was obtained from the ethical committee at Yarmouk University, Irbid, Jordan (IRB/2022/92).

## Sources of funding

The conduct of this study received no financial assistance.

## Patient consent

Informed consent was received from all participants at the beginning of the questionnaire.

## Conflicts of interest disclosure

On behalf of all the authors, the corresponding author declares no conflict of interest, including employment, consultancies, stock ownership, honoraria, paid expert testimony, patent applications/registrations, and grants or other funding.

## Research registration unique identifying number (UIN)

1. Name of the registry: Research Registry.
2. Unique identifying number or registration ID: researchregistry8717.
3. Hyperlink to your specific registration (must be publicly accessible and will be checked): https://www.researchregistry.com.

## Provenance and peer review

Not commissioned, externally peer-reviewed.
